# A phylogenetic framework for evolutionary study of the nightshades (Solanaceae): a dated 1000-tip tree

**DOI:** 10.1186/1471-2148-13-214

**Published:** 2013-09-30

**Authors:** Tiina Särkinen, Lynn Bohs, Richard G Olmstead, Sandra Knapp

**Affiliations:** 1 Department of Life Sciences, Natural History Museum, Cromwell Road, London SW7 5BD, UK; 2 Royal Botanic Garden Edinburgh, 20A Inverleith Row, Edinburgh EH3 5LR, UK; 3 Department of Biology, University of Utah, 257 S 1400 E, Salt Lake City, UT 84112, USA; 4 Department of Biology, University of Washington, Seattle, WA 98195, USA

## Abstract

**Background:**

The Solanaceae is a plant family of great economic importance. Despite a wealth of phylogenetic work on individual clades and a deep knowledge of particular cultivated species such as tomato and potato, a robust evolutionary framework with a dated molecular phylogeny for the family is still lacking. Here we investigate molecular divergence times for Solanaceae using a densely-sampled species-level phylogeny. We also review the fossil record of the family to derive robust calibration points, and estimate a chronogram using an uncorrelated relaxed molecular clock.

**Results:**

Our densely-sampled phylogeny shows strong support for all previously identified clades of Solanaceae and strongly supported relationships between the major clades, particularly within *Solanum*. The Tomato clade is shown to be sister to section Petota, and the Regmandra clade is the first branching member of the Potato clade. The minimum age estimates for major splits within the family provided here correspond well with results from previous studies, indicating splits between tomato & potato around 8 Million years ago (Ma) with a 95% highest posterior density (HPD) 7–10 Ma, *Solanum & Capsicum* c. 19 Ma (95% HPD 17–21), and *Solanum & Nicotiana* c. 24 Ma (95% HPD 23–26).

**Conclusions:**

Our large time-calibrated phylogeny provides a significant step towards completing a fully sampled species-level phylogeny for Solanaceae, and provides age estimates for the whole family. The chronogram now includes 40% of known species and all but two monotypic genera, and is one of the best sampled angiosperm family phylogenies both in terms of taxon sampling and resolution published thus far. The increased resolution in the chronogram combined with the large increase in species sampling will provide much needed data for the examination of many biological questions using Solanaceae as a model system.

## Background

Divergence times are of major interest for studies of evolutionary biology and historical biogeography, but also to researchers who focus on understanding various types of trait evolution, such as the development of chemical and genetic pathways, climatic niche and geographic range sizes, and morphological, ecological and behavioural characters. With the recent publication of fully annotated genomes in the Solanaceae [1-3], genomic tools now exist for unravelling genetic mechanisms that control such traits and their development. What is lacking, however, is a robust phylogenetic framework that encompasses species and generic diversity across the family in order to maximise the potential of these new data sources in a wider evolutionary context. Although several studies have focused on understanding evolution of particular characteristics in Solanaceae in a phylogenetic context, including analyses of genome and chromosome evolution [4-6], life history and polyploidy [7-9], floral and fruit morphology [10,11], gene family evolution and sub-functionalization [12-15], and broad-scale biogeographic patterns [16], only a single study has examined character evolution through time [8]. A central problem has been the lack of a robust, densely sampled, dated molecular phylogeny for the entire family.

Solanaceae are a particularly interesting angiosperm family, not only because they include many major crop species (e.g., potato - *Solanum tuberosum*, tomato - *S. lycopersicum*, eggplant - *S. melongena*, sweet and chili peppers - *Capsicum* spp., tobacco – *Nicotiana* spp.) and ornamentals (e.g., *Petunia* spp., *Solanum* spp.), but also a number of taxa used as biological model systems (e.g. *Nicotiana* spp., *Solanum* spp., *Petunia* spp., *Datura* spp.) [17-20]. A taxonomic and phylogenetic framework is now available for the family at the generic level (see http://www.solanaceaesource.org) [21,22]. Separate studies have explored relationships at tribal [23-26] and generic levels [27-33], and many phylogenetic studies have focused on *Solanum*, a genus that comprises nearly half of the species within the family [34-40]; see [21] for references prior to 2006]. Some of these studies have resulted in taxonomic changes, such as re-circumscription of the previously distinct genera *Lycopersicon*, *Cyphomandra*, *Normania*, and *Triguera* as parts of a monophyletic *Solanum*[35,41,42]. Such changes, although sometimes disruptive in the short term, have helped to both stabilise names and provide a better evolutionary context for future studies.

Although a relatively robust understanding of the major clades within Solanaceae exists, a densely sampled species-level phylogeny is still lacking. The most recent molecular systematic study focused on establishing major relationships within the family, but lacked depth in terms of sampling as it only included 190 (c. 7.3%) of a total c. 2,700 Solanaceae species [22]. A larger phylogenetic analysis with 995 species by Goldberg and colleagues [8] focused on the evolution of breeding systems, and did not discuss details of topology or implications for family-wide systematics. For *Solanum* itself, the most recent phylogeny included only 102 (7.7%) of the total c. 1,325 species in the genus [43]. Because several phylogenetic studies at various taxonomic levels across Solanaceae have since been published (see above), there is now a large quantity of new sequence data available for a wider family-level analysis.

Molecular divergence time analyses do not only depend on the availability of a robust, well-sampled phylogeny, but also require robust fossil calibration points [44,45]. The Solanaceae fossil record has never been fully reviewed, and only a few fossils have been used in molecular studies [8,46,47]. These studies used fossils as calibration points without a careful comparison of fossil morphology in relation to extant diversity. A recent survey of the earliest fossil record of the Asterid clade, including Solanaceae, highlighted the need to re-assess the earliest putative Solanaceae fossils that could provide robust calibration points for the crown or stem node of the family [48].

This study is part of a collaborative approach to studying the taxonomy and phylogeny of the Solanaceae. Here we present a densely sampled phylogenetic study of the family coupled with a molecular dating analysis with fossil calibrations. We review all known seed fossils in the family, and assess them for identity, age, and phylogenetic position. We then use all available sequences for seven DNA loci found in GenBank with nearly all genera and 1,075 species represented. A dating analysis is run using an uncorrelated relaxed molecular clock model within a Bayesian framework with direct fossil calibrations. The resulting time-calibrated phylogeny offers important insights into the evolution of the family at different taxonomic levels, and a robust platform for future evolutionary studies.

## Results

### Fossil review

A total of 50 fossil records previously assigned to Solanaceae were found in the literature (Table 1, see Additional file 1 for full details). These included 39 seed fossils, one leaf fossil, five flowers, two wood and three pollen fossils (Table 1). None of the leaf or flower fossils showed any distinct morphological characters that allowed us to definitely assign them to the family. Of the two wood fossils, *Solanumxylum paranensis* can be clearly assigned to Solanaceae based on a large number of anatomical characters such as para- and apotracheal axial parenchyma that is diffuse in aggregates, simple perforation plates, bordered and alternate intervessel pits, homocellular rays, fibres that are polygonal and quandrangular in section, and the presence of septate fibers (Table 1) [49]. The other wood fossil shows no specific characters of Solanaceae except those common to Solanaceae and Asteraceae and lacks axial parenchyma; we do not consider this a member of Solanaceae (Table 1) [50]. Of the two pollen records, the classification of *Datura* cf. *discolor* awaits further examination, since no description or illustration of the fossil was provided in the original publication (Table 1). A pollen fossil-taxon from California, based on two poorly preserved specimens of 3-colporate, 5-colpate, prolate shaped grains with striate ornamentation, resembles pollen grains of *Lycium*, *Nolana*, and *Hyoscyamus*[51,52]. Similar characters appear in the pollen of the unrelated genera *Brucea* (Simaroubaceae) and *Skimmia* (Rutaceae) [51,53], and hence we have not assigned this pollen fossil-taxon to Solanaceae for our analysis (Table 1).

**Table 1:** Fossil records of Solanaceae

**Name**	**Age (Mya)**	**Epoch**	**Organ**	**Fossil strata**	**Country**	**Solanaceae**	**Phylogenetic positioning**
*Datura* cf. *stramonium*	3.6 - 2.6	Pliocene: Piazencian	seed	Kholmech, Dnieper river	Belarus	Yes	Solanoideae
*Hyoscyamus* sp.	5.3 - 2.6	Pliocene	seed	Kroscienko	Poland	Yes	Solanoideae
*Hyoscyamus niger*	3.6 - 2.6	Pliocene: Piazencian	seed	Pont-de-Gail, Cantal	France	Yes	Solanoideae
*Physalis alkekengi*	7.3 - 3.6	Mio/Pliocene: Messinian-Zanclean	seed	Saugbagger Flora	France	Yes	Solanoideae
*Physalis alkekengi*	3.6 - 2.6	Pliocene: Piazencian	seed	Kholmech, Dnieper river	Belarus	Yes	Solanoideae
*Physalis alkekengi*	3.6 - 2.6	Pliocene: Piazencian	seed	Bezirk Halle	Germany	Yes	Solanoideae
*Physalis pliocenica*	28.4 - 23.0	Oligocene: Chattian	seed	Zentendorf	Germany	Yes	Solanoideae
*Physalis pliocenica*	5.3 - 2.6	Pliocene	seed	Kroscienko	Poland	Yes	Solanoideae
*Physalis pliocenica*	11.6 - 7.3	Miocene: Tortonian	seed	Stare Gliwice	Poland	Yes	Solanoideae
*Physalis pliocenica*	13.7 - 11.6	Miocene: Serravallian	seed	Tongrube Forst Neukollm	Germany	Yes	Solanoideae
*Physalis pliocenica*	13.7 - 11.6	Miocene: Serravallian	seed	Klettwitz	Germany	Yes	Solanoideae
*Physalis alkekengi*	7.3 - 3.6	Mio/Pliocene: Messinian-Zanclean	seed	Nochten-Ost	Germany	Yes	Solanoideae
*Physalis* aff. *alkekengi*	5.3 - 3.6	Pliocene: Zanclean	seed	Baldevo Formation	Bulgaria	Yes	Solanoideae
*Scopolia carniolica*	3.6 - 2.6	Pliocene: Piazencian	seed	Bezirk Halle	Germany	Yes	Solanoideae
*Solanispermum reniforme*	48.0 - 46.0	Eocene: Late Ypresian/Early Lutetian	seed	Lower Bagshot (Arne), Poole Formation	UK	Yes	Solanaceae
***Solanispermum reniforme***	**48.0 - 46.0**	**Eocene: Late Ypresian/Early Lutetian**	**seed**	**Bournemouth Freshwater Beds, Poole Formation**	**UK**	**Yes**	**Solanaceae**
*Solanispermum reniforme*	44.0 - 40.0	Eocene: Late Lutetian	seed	Boscombe Sand Formation	UK	Yes	Solanaceae
*Solanispermum reniforme*	40.4 - 37.2	Eocene: Bartonian	seed	Highcliffs sands/Cliff End Beds, Barton Formation	UK	Yes	Solanaceae
*Solanispermum reniforme*	33.9 - 28.4	Oligocene: Rupelian	seed	Bovey Tracey	UK	Yes	Solanaceae
*Solanum arnense*	48.0 - 46.0	Eocene: Late Ypresian/Early Lutetian	seed	Lower Bagshot (Arne), Poole Formation	UK	Yes	Solanaceae
*Solanum* cf. *persicum*	3.6 - 2.6	Pliocene: Piazencian	seed	Kholmech	Belarus	Yes	Solanoideae
*Solanum dulcamara*	7.3 - 3.6	Mio/Pliocene: Messinian-Zanclean	seed	Saugbagger Flora	France	Yes	Solanoideae
*Solanum dulcamara*	3.6 - 2.6	Pliocene: Piazencian	seed	Bezirk Halle	Germany	Yes	Solanoideae
*Solanum dulcamara*	3.6 - 2.6	Pliocene: Piazencian	seed	Nordhausen	Germany	Yes	Solanoideae
*Solanum dulcamara*	3.6 - 2.6	Pliocene: Piazencian	seed	Rippersroda	Germany	Yes	Solanoideae
*Solanum dulcamara*	5.3 - 2.6	Pliocene	seed	Tegelen-Sur-Meuse	Holland	Yes	Solanoideae
*Solanum dulcamara*	3.6 - 2.6	Pliocene: Piazencian	seed	Pont-de-Gail, Cantal	France	Yes	Solanoideae
*Solanum dulcamara*	3.6 - 2.6	Pliocene: Piazencian	seed	Pont-de-Gail, Cantal	France	Yes	Solanoideae
*Solanum dulcamara*	5.3 - 2.6	Pliocene	seed	Kroscienko	Poland	Yes	Solanoideae
*Solanum nigrum*	11.6 - 5.3	Miocene: Tortonian-Messinian	seed	North Rhine, Hambach	Germany	Yes	Solanoideae
*Solanum* sp.	5.3-2.6	Pliocene	seed	Limburg & Prussian border	Holland	Yes	Solanoideae
***Cantisolanum daturoides***	**55.0 – 50.0**	**Eocene: early Ypresian**	**seed**	**London Clay Formation**	**UK**	**No**	**-**
Unknown Solanaceae	5.3-2.6	Pliocene	seed	Limburg & Prussian border	Holland	No	-
*Physalis* sp.	5.3 - 0.0	Plio/Pleistocene	seed	Torre Picchio section (P11 & P12), Santa Maria di Ciciliano Formation	Italy	? ^1^	-
*Solanum* sp.	1.7 - 1.2*	Pleistocene	seed	Olduvai Gorge	Tanzania	? ^1^	-
*Physalis* sp.	23.0 - 16.0	Miocene: Aquitanian-Burdigalian	seed	Kireevsky Village, Ob River	Russia	? ^2^	-
*Solanum* sp. #1	3.6 - 2.6	Pliocene: Piacenzian	seed	Chernoluch Village, Irtysh River	Russia	? ^2^	-
*Solanum* sp. #2	16.0 - 11.6	Miocene: Langhian-Serravallian	seed	Novonikolsky Village, Irtysh River	Russia	? ^2^	-
*Solanum* sp. #2	16.0 - 11.6	Miocene: Langhian-Serravallian	seed	Ebargulsky Village, Irtysh River	Russia	? ^2^	-
*Solanumxylon paranensis*	16.0 - 11.6	Miocene: Langhian-Serravallian	wood	Paraná Formation	Argentina	Yes	Solanaceae
Solanaceae or Asteraceae	70.6 - 65.5	Cretaceous: Maastrichtian	wood	Panoche Formation, Del Puerto	USA	No	-
Pollen forma C	72.0 - 68.0	Cretaceous: Campanian-Maastrichtian boundary	pollen	San Joaquin Valley (D-1 & D-2), Great Valley Sequence	USA	No	-
*Datura* cf. *discolor*	37.2 - 33.9	Eocene: Priabonian	pollen	Florissant Basin	USA	? ^1^	-
*Solanum* sp.	5.3 - 0.0	Plio/Pleistocene	pollen	Olduvai Gorge	Tanzania	? ^1^	-
*Solandra haeliadum*	55.8 -33.9	Eocene	leaf	Salcedo	Italy	No	-
*Solanites brongniartii*	33.9 - 23.0	Oligocene	flower	Aix-en-Provence	France	No	-
*Solanites crassus*	55.8 - 40.4	Eocene: Lutetian-Ypresian	flower	Claiborne	USA	No	-
*Solanites pusillus*	55.8 - 40.4	Eocene: Lutetian-Ypresian	flower	Claiborne	USA	No	-
*Solanites saportanus*	55.8 - 40.4	Eocene: Lutetian-Ypresian	flower	Claiborne	USA	No	-
*Solanites sarachaformis*	55.8 - 40.4	Eocene: Lutetian-Ypresian	flower	Claiborne	USA	No	-

List of all known fossil records of Solanaceae. Fossils highlighted in bold were scanned using high-resolution X-ray computed tomography [T.Särkinen, M.Collinson, P.Kenrick, F.Ahmed, unpublished observations]. For full details of the fossil and references to primary sources, see Additional file 1.

^1^ No description nor illustration or proper reference to specimen.

^2^ Reference not located.

The putative Solanaceae seed fossils were analysed using a combination of characteristics known from clades within the family [54]: (1) Seeds flattened, (2) circular to reniform in shape, (3) hilum sub-laterally or laterally positioned, and (4) testa cells sinuate-margined. We assigned seeds with all four of these characters to the subfamily Solanoideae (N = 28), while those with some but not all of these were assigned to the family as a whole (N = 6) (Table 1) [10]. The currently recognised earliest fossils assignable to Solanaceae include two seed fossils from Eocene Europe: *Solanispermum reniforme* recorded from various beds from southern England [55,56], and *Solanum arnense*, a fossil-taxon described based on a few specimens found from the Lower Bagshot (Table 1) [55]. Neither of these shows the combination of flat seeds with sinuate-margined testa cells, a unique combination that could tie them to the tribe Solanoideae. The flattened seeds of *Solanispermum reniforme* lack sinuate margined testa cells, and *Solanum arnense* seeds show the characteristic testa cells but seeds are round rather than flattened. Hence, we consider these fossils as earliest evidence of Solanaceae and the presence of the family in Eocene Europe, but do not assign them to any particular clade within the family. Seeds of the fossil-taxon *Cantisolanum daturoides*[57] have previously been cited as the oldest known Solanaceae fossil by some authors [10] but doubtfully a member of the family by others [48]. Results from a CT-scanning study have shown that this *Cantisolanum* seed is anatropous and does not belong to Solanaceae, but has likely affinities to the monocot family Philydraceae [T. Särkinen, M. Collinson, P. Kenrick, F. Ahmed, unpublished observations].

### Solanaceae phylogeny

Our final supermatrix had a taxon coverage density of 0.45, and included 1,075 species of Solanaceae, representing all but two genera (the monospecific *Darcyanthus* and *Capsicophysalis*) and 40% of total species within the family, including 34% sampling of species within the large genus *Solanum*. Two plastid regions, *ndhF* and *trnL-F*, were available for all genera except *Darcyanthus* and *Capsicophysalis* and the plastid and nuclear regions ITS, *waxy* and *trnL-F* were the most densely sampled regions at the species level (Table 2). The matrix included a total of 4,576 variable characters, with an aligned length of 10,672 bp (Table 2). A total of 1,902 bp were excluded from analyses due to ambiguous alignment (see Methods section) resulting in a matrix of 8,770 bp (Table 2). Proportionately, *waxy* (33.9%) and *ndhF* (20.6%) contributed most PI (parsimony informative) characters (Table 2). The relatively little-used plastid region *trnS-G* showed a surprising number of PI characters (13.5% of total), considering it had relatively poor taxon coverage density (0.23), compared to *trnL-F* which had a coverage of 0.66 but only 6.6% of total PI characters (Table 2). The final matrix included 54.7% missing data (Table 2). At the species level, there was an average of 58.7% missing data, as measured by number of base pairs, but only 49.9% when measured in terms of PI characters expected from the missing regions.

**Table 2:** Supermatrix details

**Region**	**Genome**	**Genera**	**Species**	**Aligned length**^**1**^	**Variable characters**	**%**	**PI characters**	**%**	**PI of combined matrix (%)**	**Missing data (%)**	**Taxon coverage density**
*ITS*	Nuclear ribosomal	51	765	585	461	78.8	345	59.0	10.6	28.8	0.71
*matK*	Plastid	44	287	1027	419	40.8	262	25.5	8.1	73.3	0.27
*ndhF*	Plastid	90	472	2109	960	45.5	670	31.8	20.6	56.1	0.44
*psbA-trnH*	Plastid	30	223	698	340	48.7	227	32.5	7.0	79.3	0.21
*trnS-G*	Plastid	15	246	1329	694	52.2	439	33.0	13.5	77.1	0.23
*trnL-F*	Plastid	90	707	905	353	39.0	206	22.8	6.3	34.2	0.66
*waxy*	Nuclear	41	708	2085	1349	64.7	1104	52.9	33.9	34.1	0.66
**Total**		90	1075	8738	4576	52.4	3253	37.2	100	54.7	0.45

List of the seven regions used for building the Solanaceae mega-phylogeny, showing level of sampling at specific and generic levels, aligned length, and proportions of parsimony informative (PI) and variable sites per region. Values have been calculated without outgroups.

^1^ingroup only, excluded regions omitted.

**Figure 1:**
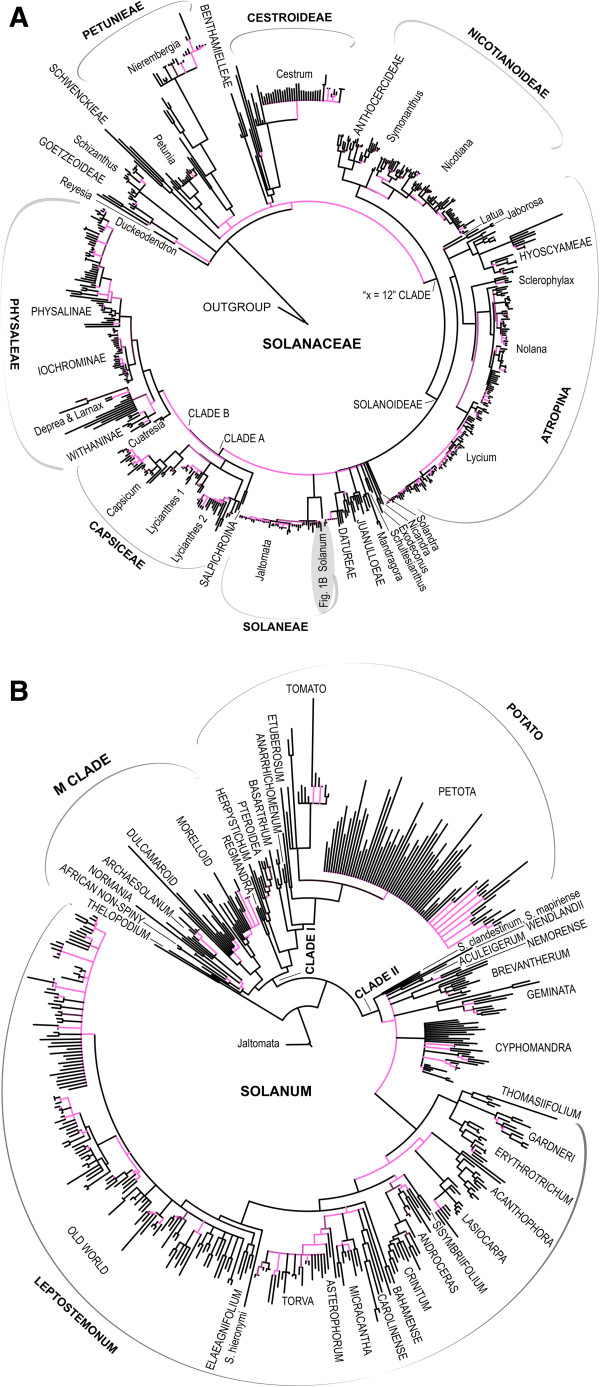
**Solanaceae phylogeny.** Phylogenetic relationships between major clades of Solanaceae based on a Maximum Likelihood analysis of a 1076 taxon supermatrix (ITS, *waxy*, *ndhF*, *matK*, *psbA*-*trnH*, *trnS*-*G*, *trnL-F*) with 10,672 bp of sequence data. Major clades recovered by previous phylogenetic studies [22,43,64] are labelled, as is the M Clade identified for the first time here. Clades with low bootstrap support (60-79%) are shown in pink, while strongly supported clades (boostrap support 80-100%) are in black. **A**. Major clades of Solanaceae. **B**. Relationships within *Solanum*.

The resolved Maximum Likelihood topology shows strong support for all previously identified major clades within Solanaceae [22], and increased node support is observed particularly within *Solanum* (Figure 1). Only major clades and their relationships are discussed here due to the fact that our analyses only accounted for incongruence issues amongst data sets between major clades rather than at shallow taxonomic levels. We encourage readers to refer back to available clade-specific studies for detailed species-level phylogenies (see references cited here and in ref. [21] for studies prior to 2006); these studies have incorporated larger sets of markers than used here, incorporate methods that test/account for gene tree – species tree incongruence, and discuss issues that could have led to any detected incongruences between gene trees such as polyploidy and/or hybridisation, and incomplete lineage sorting.

The branching order at the base of Solanaceae is not well defined, similar to the findings of Olmstead et al. [22], and four groups are identified as the first branching taxa: *Schizanthus*, *Duckeodendron,* the previously unplaced *Reyesia*, and the tribe Goetzeoideae (Figure 1). *Reyesia* has been previously associated with *Salpiglossis*[54], but is here placed with Goetzeoideae and *Duckeodendron* (Figure 1, Additional file 2). The previously unsampled genera *Heteranthia, Trianaea* and *Schraderanthus* are placed within Schwenckieae, Juanulloeae, and Physalinae, respectively (Figure 1, Additional file 2). The informally named X = 12 clade is here recovered with strong support and Nicotianoideae is resolved as sister to the rest of the clade (Figure 1). Within the Physalinae, work is clearly needed to delimit monophyletic genera (Figure 1, Additional file 2, see [58,59]). Two closely related genera, *Larnax* and *Deprea*, are resolved as sister to Withaninae, in agreement with morphology (Figure 1, Additional file 2). These genera have been linked with Iochrominae in some molecular analyses [22], but considered distant outgroups of Iochrominae by others [58,59]. The molecular data support the treatment of *Schraderanthus* as distinct from *Leucophysalis*[60], and *Schraderanthus* is here found as sister to *Brachistus* + *Witheringia* (Figure 1, Additional file 2).

Within *Solanum*, all 12 major clades identified by Weese & Bohs [43] are recovered, with nearly fully resolved relationships among them (Figure 1). The Thelopodium clade is resolved as the first branching group, and the remaining *Solanum* species are divided into two strongly supported clades. Clade I comprises all non-spiny, often herbaceous (e.g., tomatoes, potatoes) species without stellate hairs, but also includes woody climbers (e.g., Dulcamaroids) and some shrubby species (e.g., Morelloids). Clade II comprises species that are often shrubs or small trees (although some are only weakly woody), many with prickles and/or stellate hairs (Figure 1). Within Clade I, which includes a total of c. 525 known species, two clear clades are resolved: (1) the Potato clade, with Regmandra clade as the first branching group, and (2) Clade M, including Morelloid, Dulcamaroid, Archaesolanum, Normania, and the African Non-Spiny clades (Figure 1). Relationships within Clade M are well resolved and highly supported, revealing the position of the African Non-Spiny clade as distinct from and not closely related to the Dulcamaroid clade, despite their morphological similarities such as a twining habit and twisting petioles [61]. Within the Potato clade, relationships are equally well resolved: section *Petota* is resolved as sister to a group comprising the Tomato clade plus a set of smaller early-branching clades (Figure 1). The Regmandra clade, a group of 11 species whose centre of diversity is the hyper-arid Atacama desert, is here resolved as part of the Potato Clade for the first time (Figure 1), a result supported by morphology [62,63].

Relationships within Clade II are less well-resolved. The clade consists of c. 800 mostly woody species, and includes the large Leptostemonum clade known as "spiny solanums". There is moderate support for *S. clandestinum* + *S. mapiriense* as sister to the rest of Clade II (Figure 1). Relationships within the large Leptostemonum clade remain relatively unresolved, but all 14 major clades found in previous analyses [64] are supported. A set of previously unplaced species, *S. crotonoides*, *S. hayesii*, and *S. multispinum*, are resolved sequentially as sister to the Torva clade (Additional file 2), although on morphological grounds *S. hayesii* would be a member of the Torva clade.

### Molecular dating

The general topology of the Bayesian maximum clade credibility tree matched that of the best scoring Maximum Likelihood tree with similar levels of support for major clades (Additional file 3). The only topological difference, although not a hard incongruence, was observed at the base of Solanaceae: Bayesian analyses resolved Schwenckieae as the first branching group within the family, while the base of the tree remained largely unresolved in the maximum likelihood topology. Results from PATHd8 gave generally similar ages as those from the BEAST analysis (Table 3). A notable trend is that BEAST ages were consistently younger especially towards the early-branching nodes (Table 3). The younger ages obtained from the BEAST analysis reflect that diversification rates across Solanaceae have been non-linear especially towards the base of the tree, and/or that extinction and speciation rates have varied across the tree. We will focus our discussion on the BEAST results, which we consider to be more robust due to the more realistic model assumptions used, including the relaxed molecular clock model that accounts for rate variation across lineages, as well as Birth-Death tree model accounting for extinction [65].

**Table 3:** Molecular age estimates

**Clade**	**Group members**	**Dating method**	
		**BEAST (uncorrelated lognormal relaxed clock model)**	**PATHd8 (local clock model)**
Tomato-potato	*S. lycopersicon* – *S. tuberosum*	8.0 (6.6-9.5)	9.1
Eggplant-tomato/potato	*S. lycopersicon* – *S. melongena*	14.3 (12.5-16.3)	18.8
Section *Lycopersicon*	*S. lycopersicon* – *S. peruvianum*	2.0 (1.2-2.6)	2.9
Section *Petota*	*S. tuberosum* – *S. piurae*	7.1 (5.9-8.5)	7.5
Eggplant clade	*S. melongena* – *S. macrocarpon*	3.4 (2.7-4.1)	2.8
*Capsicum* cultivated	*C. frutescens* – *C. eximium*	3.4 (2.7-4.3)	3.6
*Solanum* Clade I – Clade II	*S. melongena* – *S. tuberosum*	14.3 (12.5-16.3)	18.8
*Solanum* (crown)	*S. melongena* – *S. thelopodium*	15.5 (13.3-17.5)	22.0
*Solanum* (stem)	*Solanum* – *Jaltomata*	17.0 (14.5-18.7)	23.6
*Solanum*-*Capsicum*	*Solanum* – *Capsicum*	19.1 (17.0-21.0)	25.6
Solanoideae	*Solanum* – *Lycium*	21.0 (19.0-23.3)	25.6
*Solanum*-*Nicotiana* (i.e., X = 12 clade) (i.e., Solanoideae stem)	*Solanum* – *Nicotiana*	24.0 (23.0-25.7)	28.4
Solanaceae (crown)	*Solanum* – *Schizanthus*	30.4 (26.3-34.0)	32.1
Solanaceae (stem)	Solanaceae - Convolvulaceae	49.2 (46.2-53.7)	54.8

Minimum age estimates produced based on both PATHd8 and BEAST analyses for Solanaceae and its major clades. Clades refer to crown nodes unless otherwise indicated. Ages in brackets are 95% confidence intervals for BEAST. A densely sampled, time calibrated Solanaceae mega-phylogeny with associated confidence intervals can be found in Additional file 2.

The BEAST results place the stem age of Solanaceae at c. 49 Million years ago (Ma, 95% highest posterior density (HPD): 46–54 Ma), and the crown node at c. 30 Ma (95% HPD 26–34) (Additional files 2 and 3). The crown node of the x = 12 Clade, which is the split between *Nicotiana* and *Solanum*, was estimated to be c. 24 My old (95% HPD 23–26) (Additional files 2 and 3). The Solanoideae began diversifying c. 21 Ma (95% HPD 19–23) (Additional files 2 and 3). *Solanum*, a genus which includes nearly half of the total species diversity in the family, split from *Jaltomata* c. 17 Ma (95% HPD 15–19) and started diversifying c. 16 Ma (95% HPD 13–18) (Additional files 2 and 3). The *Solanum* – *Capsicum* split, corresponding to the most common ancestor of *Solanum & Physalis*, occurred c. 19 Ma (95% HPD 17–21) (Additional files 2 and 3). Within *Solanum*, major splits include tomato – potato c. 8 Ma (95% HPD 7–10) and the eggplant –tomato/potato lineages corresponding to the Clade I – Clade II split c. 14 Ma (95% HPD 13–16) (Additional files 2 and 3). Crown node age estimates show that section *Petota*, which includes all cultivated potatoes, started diversifying c. 7 Ma (95% HPD 6–9), section *Lycopersicon*, which includes the cultivated tomato, c. 2 Ma (95% HPD 1–3), the group containing all species of cultivated eggplants (*S. melongena, S. anguivi* and *S. macrocarpon*) c. 3 Ma (95% HPD 2–4), and the group (*C. frutescens* – *C. eximium*) including all cultivated pepper species c. 3 Ma (95% HPD 2–4) (Additional files 2 and 3).

## Discussion

### Phylogenetic relationships within Solanaceae

Although individual studies have contributed significantly to a better understanding of the systematics and evolution of the family at generic and tribal levels, our results bring together data from a large number of studies into a single analysis, and present a coherent view on the current systematic knowledge of this diverse family and its major clades. Our analyses support all of the major clades previously identified within Solanaceae [22], *Solanum*[43] and the Leptostemonum clade of *Solanum*[64]. All of these major clades within the family are now strongly supported, and furthermore, our results reveal strongly-supported relationships between the major clades of the mega-diverse genus *Solanum*, strengthening the backbone.

The increased resolution in the current phylogeny can be attributed to both the increased sampling of markers as well as species. In the quest for better resolved phylogenies, studies often seek large amounts of sequence data, but it is now well established that increased species sampling can have an equally positive effect on phylogenetic resolution and accuracy [66-68]. The addition of more species to a data set has the effect of splitting long branches and detecting multiple substitutions, as well as resolving phylogenetic conflict, improving parameter estimation, and making inferences less dependent on particular evolutionary models [68]. In our approach we chose to maximise species sampling, while minimising missing data by choosing only the most densely sampled markers available. This approach generally boosted resolution without introducing any of the significant negative effects that large amounts of missing data can have on phylogeny estimation.

Our study presented here is a significant step forward in working towards a fully sampled species-level phylogeny for Solanaceae. A previous study by Goldberg et al. [8] included 995 species but did not present a fully annotated molecular phylogeny that would allow an analysis of systematic relationships within the family. With > 1,000 species now covered, the current phylogeny includes 40% of known species and all genera of Solanaceae, except the monospecific and recently segregated *Darcyanthus* and *Capsicophysalis*. This is a substantial improvement on previous studies, and our current phylogeny is one of the best sampled family-level studies in angiosperms e.g., [69-71].

The sampling is now adequate to test for generic monophyly in previously poorly sampled groups. Although the number of genera is becoming stable with 97 currently recognised genera in Solanaceae (recent changes include those documented in refs. [26,60,72]), our analyses support previous results in identifying a set of groups where generic re-evaluation will be necessary, including *Lycianthes*/*Capsicum*, the genera in the Physalineae (especially *Physalis*) [59], *Deprea*/*Larnax*, the Iochrominae [58], and the Australian endemics in the Anthocercideae (see Additional file 2); many of these clusters of generic problems have been identified by previous authors.

Broader level relationships within Solanaceae and *Solanum*, as well as generic delimitations and problems identified in previous studies are supported by our species-rich dataset. Relationships between some of the major clades remain unresolved, however, most notably those at the base of the family and within the Solanoideae, and the Leptostemonum clade of *Solanum*. Resolving these nodes will be a priority in order to better understand evolution of some particularly complex traits, such as chromosome evolution. For example, resolving the sister group to the X = 12 clade, as well as the first branching taxa within Solanaceae, would allow us to determine the ancestral base chromosome number in the family and to fully understand directionality of chromosome evolution. Despite the increased resolution introduced by the use of more sequence data and higher species-level sampling, our results do not show any improvement in the resolution in these critical nodes. More genes will be needed to resolve these relationships, but the question remains which genes should be used. Highly variable nuclear loci, such as COSII markers already used in Solanaceae [73,74], and the PPR genes used in families within the related Asterid order Lamiales [75,76], present the most promising candidates. The widely sequenced regions ITS, *waxy, ndhF*, and *trnSG* are the most variable across the Solanaceae and species-level sampling using these regions should be increased. The traditionally used plastid marker *trnT-F*, which is relatively slowly evolving within Solanaceae, is known to include pseudogenes in *Solanum*[77] and care should be taken when using this region in phylogenetic studies.

### Solanaceae fossil record

A few fossils have been used in previous molecular dating studies of Solanaceae, but without re-evaluation of fossil morphology and hence their placement within the phylogeny [29,46]. As revealed by our literature review, a relatively large record for the family exists. The most usable evidence comes from fossil Solanaceae seeds, the oldest of which are from Eocene Europe (c. 48–40 Ma), with a sharp increase in the number of seed morphotypes observed towards the Pleistocene. The fossil seeds can be divided into two sets: (1) seeds showing four morphological characters present in the extant members of the Solanoideae, and (2) seeds that bear resemblance to the family in general but cannot be assigned to more specific clades within it because they lack the unique combination of seed flattening and presence of sinuate-margined testa cells. Although some of these fossils have been described with names associated with extant species and/or genera, our morphological review shows that none of them show unique morphological characters that can be used to place them to any extant genera. We consider the placement of these fossils on terminal nodes as has been done by previous authors [29,46] unjustified.

All of the fossils we were able to unambiguously identify as Solanaceae are from Eocene Europe, where none of the first branching lineages of the family occur. South America is the centre of diversity of extant Solanaceae, and all of the early diverging lineages are exclusively found in the New World. This suggests that the fossil record of the family is still far from complete, and that further studies on South American fossils might reveal crucial evidence with respect to the timing of diversification in Solanaceae. A promising avenue for future fossil studies would be to carefully evaluate wood fossil records, especially Cretaceous-Eocene material from the area in which the early-branching lineages all now occur [16,22].

### Dates for Solanaceae

Our study is the largest Bayesian molecular dating analysis executed to date in terms of taxon sampling. Most previous studies have used Bayesian dating methods after pruning their original, large phylogenetic datasets largely due to an a priori assumption that Bayesian methods cannot cope with datasets with >500 terminals e.g., [78-80]. Our study with 1,075 species and >10,000 bp of sequence data demonstrates that large matrices with >500 terminals can be analysed using Bayesian dating methods. Further studies are needed, however, to fully explore best methods for analysing large datasets with the currently available dating methods that implement relaxed molecular clock models required for analyses of diverse clades where rates are expected to vary [81,82]. Such studies should focus on exploring trade-offs between number of taxa, complexity of models and partitions used in order to fully understand limitations and potential error sources in large scale analyses.

In our dating analysis, we followed the recent recommended best practice guidelines for fossil calibration [83] and placed fossil calibrations at stem nodes of the most inclusive extant groups using apomorphy-based morphological assignment. Morphological evidence from the seed fossils only allowed assignment to the broad groups Solanoideae or Solanaceae as a whole. Fossils provide only minimum age estimates for the nodes they are assigned, and hence results from our dating analysis where fossil calibrations were used should be considered as minimum age estimates. We further biased our results towards younger ages by assigning the oldest known fossils of Solanaceae to the stem node of the family rather than to more specific nodes within Solanaceae due to lack of morphological and anatomical characters that could be used to assign them to more specific nodes. There is always a possibility, however, that these seeds represent more specific clades within Solanaceae, which would push back age estimates for the family. Currently, the earliest fossil evidence for the family comes from Eocene Europe, but based on biogeographic analyses, the crown group of Solanaceae is thought to have originated and first diversified in South America [16,22,84]. Total evidence analysis, where fossils are placed as terminal taxa in the dating analysis using both molecular and morphological data matrix, could help in exploring the robustness of fossil placement [85], but as pointed above, the lack of characters in the Solanaceae seed fossils does not currently permit such analyses. The most promising avenue in strengthening the dating analysis would be in finding further fossil records (see Solanaceae fossil record above). This would increase the number of fossil calibration points and allow the use of cross-validation methods [86].

The rate of molecular evolution in plants has been found to correlate with life history traits, whereby longer living species show consistently lower substitution rates compared to shorter living species [81]. Molecular clock models should incorporate such rate variation, especially in groups such as Solanaceae which include a range of growth and life forms. Our dating analyses did not incorporate such models, although the model used in our Bayesian analysis allows rates to vary between lineages independently. The lack of such models in our analyses implies that the age of herbaceous, shorter lived plants (e.g., *Schizanthus* and the Tomato clade of *Solanum*) will be systematically overestimated, while ages in dominantly woody clades (e.g. *Solanum* Clade II) will be consistently underestimated. Future studies should explore how molecular clock models that account for rate variation due to life history traits could be implemented.

Previous studies have produced a wide range of estimates for the stem age of the family, ranging from 34–85 Ma [48,87-89], but none of these studies included dense sampling within the family nor used robust Solanaceae-specific fossil calibrations. Paape et al. [90] analysed divergence times within Solanaceae but with a small dataset consisting of 29 species only. This study was based on three fossil calibration points without re-assessment or morphological study of the original fossils, and estimated Solanaceae stem age to have diverged 62 Ma (95% HPD 54–70 Ma) [90]. The oldest estimates for the family stem node age come from earlier molecular studies which used calibration points with more simplistic dating methods (65–85 Ma) [87,88], while the most recent molecular dating study of angiosperms by Bell et al. [89] who used 36 fossil calibrations across the tree and a relaxed molecular clock model, estimated the Solanaceae stem node to have diverged c. 59 Ma (95% HPD 49–68 Ma). Our results, which we consider as minimum ages, are broadly consistent with Bell et al. [89] in estimating the stem node of Solanaceae to date back to c. 49 Ma (95% HPD: 46–54).

The age of the major splits within the family has been of interest to various fields, including studies on chromosomal [4] and genome evolution [5,6,91]. Our minimum age estimates for the major splits between tomato – potato (c. 8 Ma, 95% HPD 7–10), eggplant – tomato/potato (c. 14 Ma, 95% HPD 13–16), *Solanum* – *Capsicum* (c. 19 Ma, 95% HPD 17–21), and *Solanum* – *Nicotiana* (c. 24 Ma, 95% HPD 23–26) are consistent with the age estimates produced in previous studies without fossil calibrations using much sparser sampling and more simplistic molecular clock models [4,6,91]. Our results for the *Nicotiana* – *Symonanthus* split (c. 15 Ma, 95% HPD 11–20) corroborate results obtained using island age (c. 15 Ma) [92] and those calculated using paralogy-free subtree analysis (>15 Ma for section *Suaveolentes*) [93]. Our results presented here suggest that the rate of chromosomal and genome evolution within Solanaceae has been marginally slower at least within particular lineages than previously thought. With the densely sampled chronogram presented in this study, a more detailed analysis of chromosomal evolution at the species level could now be performed in the Solanaceae to study rate differences and drivers of chromosomal changes such as environmental or life history factors. Similarly, morphological characters such as fruit type [10] could be analysed in relation to diversification rates to identify whether particular morphological traits are associated with speciation rate shifts in Solanaceae.

## Conclusions

Despite much focus on character and trait evolution within Solanaceae, little has been known about the origin of traits in the family in terms of time. We present here minimum age estimates and associated confidence intervals for the entire Solanaceae using a species-rich dataset comprising almost half of the species diversity within the family. This densely sampled chronogram will provide the basis for unravelling the tempo and mode of evolution of many of the much-studied and complex traits in this diverse and economically important family such as self-incompatibility, fruit type, cold and salt tolerance, disease resistance, chromosomal re-arrangements, genome size, and gene sub-functionalization.

## Methods

### Fossil study

References to fossil records were compiled from various sources, including Yale Paleobotany Online Catalog (http://peabody.yale.edu/collections/paleobotany), the Paleobiology Database (http://paleodb.org), InsideWood Database (http://insidewood.lib.ncsu.edu), Burke Paleontology Collection Database (http://www.washington.edu/burkemuseum/collections/paleontology), the Stratigraphy Database (http://www.stratigraphy.net), Fossil Record 2 [94], and Google searches on terms "Solanaceae" and "fossil". The morphology of two fossil specimens was analysed using high-resolution X-ray computed tomography (Table 1) [T. Särkinen, M. Collinson, P. Kenrick, F. Ahmed, unpublished observations]. The morphology of other specimens was evaluated using descriptions and illustrations provided in original publications. The numeric ages for fossils were derived by matching the specific strata from which fossils were found with the most recent geochronological stratigraphy found in the literature (see Additional file 1). The oldest fossil specimens assigned to Solanaceae and the Solanoideae stem nodes were then used as calibration points (see below). The younger age brackets of these oldest specimens were used following best practise guidelines [83].

### Supermatrix construction and analysis

Our supermatrix data harvesting and construction largely followed the modified supermatrix method termed 'mega-phylogeny’ designed for larger datasets by Smith et al. [95]. The mega-phylogeny method has been designed for large datasets, where maximally dense supermatrices are built based on BLAST searches of all genebank sequences limited to the taxonomic rank of interest [95]. This differs from traditional supermatrix approach where no threshold to missing data or taxa is set, and the resulting sparser matrices are built using clustering techniques.

We looked for all orthologous sequence data available in GenBank release 184 using the PhyLoTA Browser [96]. PhyLoTA identifies available sequence clusters based on BLAST searches, where all sequences for the specified taxonomic group are blasted against each other. We explored all phylogenetically informative sequence clusters identified by PhyLoTA for Solanaceae, and chose seven clusters that had the highest taxon sampling both in terms of genera and species. These seven clusters included data from two nuclear (*waxy* and ITS) and five plastid regions (*matK*, *ndhF*, *trnS-G*, *trnL-F*, *psbA-trnH*) (Table 2). Gaps in generic sampling were identified and sequences for three previously unsampled genera, *Trianaea*, *Heteranthia*, and *Archihyoscyamus*, were generated for *ndhF*, *trnL-F*, and ITS (Additional file 4). Further sequences were generated for poorly sampled genera (*Reyesia*, *Benthamiella*, *Deprea*, and particular clades of *Solanum*) (Additional file 4). The new sequences were joined with the clusters downloaded from PhyLoTA. Each region was aligned using the profile alignment algorithms Muscle [97] and MAFFT [98,99], after which all datasets were manually checked and adjusted to assure high quality alignments. MAFFT produced better quality alignments compared to Muscle for the most complex alignments (ITS and *waxy*) based on visual comparisons. Short multirepeats and ambiguously alignable regions were excluded. For *trnL-F*, a variable repeat region towards the 5’ end of the intergenic spacer was removed; this is where putative pseudogenic copies of *trnF* have been found in *Solanum*[77]. Taxon names were checked for synonomy in all matrices. Duplicate sequences for species were pruned out*. Montinia* (Montiniaceae), *Convolvulus* and *Ipomoea* (Convolvulaceae) were added as outgroups representing two of the closely related families of Solanaceae within the order Solanales [100] Gene regions were analysed individually using MrBayes v. 3.1.2 [101,102] via the Oslo Bioportal [103] in order to visually check for topological incongruence, rogue taxa, and presence of potentially misidentified sequences.

Ten potentially misidentified sequences were detected in the individual analyses and removed prior to supermatrix construction (Additional file 5). No hard incongruences were detected between the individual matrices with respect to the major clades of the Solanaceae. Incongruence issues were not tested at shallower taxonomic levels due to methodological constraints, and hence individual studies cited in the Background section should be referred to for phylogenetic relationships within genera or major clades in *Solanum*. The software AIR-Appender as implemented in the Oslo BioPortal [103] was used to concatenate the individual matrices. We measured missing data in two ways: missing data per gene region and per species. Missing data for each species was calculated using two measures, missing data and missing information. Missing data was measured as the absolute number of missing base pairs, while missing information was measured as the sum of the parsimony informative characters of missing regions. All species with > 90% missing data and/or information were removed prior to analysis.

Before analysis, the matrix was cleaned by pruning rogue taxa, identified as unstable terminals causing artificial lowering of branch support, using the software RogueNaRok [104]. RogueNaRok analyses were based on trees derived from fast RAxML bootstrap analyses using a 50% majority-rule consensus threshold and support values for optimization with drop setsize set to one. Four iterations were run and rogue taxa were removed after each iteration. Rapid bootstrap analyses were run in RAxML-VI-HPC v2.0.1 [105,106] via the CIPRES Science Gateway [107] applying partitioning for each gene region using a GTR + CAT approximation rate substitution model and the rapid Bootstrap algorithm with 100 replicates [106]. We removed a total of 85 rogue taxa, some of which had a large amount of missing data and/or information (60-90%), but others with nearly complete sampling. The final matrix included 10,672 bp of aligned sequence data of which 1,902 bp were excluded due to ambiguous alignment (Additional files 6, 7, 8). The matrix included a total of 1,075 Solanaceae species and a single outgroup (*Ipomoea*, Convolvulaceae). We minimized outgroup sampling in order to simplify the BEAST analysis, as the number of outgroups significantly affected run time. The final supermatrix was analysed using RAxML-VI-HPC v2.0.1 [105] via the CIPRES Science Gateway applying partitioning for each gene region using GTR + CAT approximation rate substitution model and the rapid Bootstrap algorithm with 1,000 replicates. The resulting trees were used either as input trees or as starting topologies for dating analyses.

### Molecular dating analyses

The Bayesian uncorrelated relaxed clock-model as implemented in BEAST [108,109] was used as a primary dating method because it allows for rate variation across branches and measures for rate autocorrelation between lineages. Topology and node ages are estimated simultaneously in BEAST, hence topological uncertainty is incorporated into node age estimation. The best tree from the RAxML search was used as a starting topology (Additional file 9). Each region was partitioned separately and given its own substitution model (GTR + G) and rate. A Birth-Death tree prior was used, which accounts for both speciation and extinction [110]. The Solanoideae seed fossils were used to constrain the stem node of Solanoideae with a lognormal offset of 23.0 Ma, mean of 0.01, and standard deviation (SD) of 1.0. The age constraint reflects the youngest age bracket of the oldest known fossil seed assignable to the Solanoideae. Similarly, the Solanaceae stem node was constrained with a lognormal offset of 46.0 Ma, mean of 0.01, and SD of 1.0 based on the youngest age estimate of the oldest fossil specimen of Solanaceae type seeds. Priors for the relaxed clock model mean rate and standard deviation were set to 1.0 and 0.3, respectively, based on known substitution rates in plants. The parameter weights of the delta exchange operator were modified to reflect the length of each partition. Default priors were used for all other parameters. A total of 100 million generations (10 runs with c. 10 million generations each) were run in BEAST v.1.7.4 [108]. Results were combined using LogCombiner and TreeAnnotator (BEAST package).

A second dating analysis was run using PATHd8 [111]. PATHd8 is a local rate smoothing method that estimates node ages by calculating mean path lengths from the node to the tips. Deviations from a strict molecular clock are corrected as suggested by the calibrated nodes. Only simple calibrations are allowed as point estimates of minimum, maximum or mean ages. Because substitution rates are smoothed locally, rather than simultaneously over the whole tree, PATHd8 allows analysis of very large trees. The best tree from the RAxML search was used as the input phylogeny for the PATHd8 analysis (Additional file 10). The stem node of Solanoideae was constrained with the identified Solanoideae seed fossils with minimum age of 23.0 Ma. PATHd8 requires a minimum of one fixed node constraint, and hence the stem node of the family was constrained with a fixed age of 46.0 Ma. Results from both the Maximum Likelihood and Bayesian dating analyses have been deposited in TreeBase (http://purl.org/phylo/treebase/phylows/study/TB2:S14458).

## Competing interests

The authors declare that they have no competing interests.

## Authors’ contributions

TS carried out the molecular sequence alignments and analyses, and the fossil review. LB provided sequence data for missing and poorly sampled genera. RO participated in the sequence alignment. SK participated in the design and coordination of the study. All authors contributed to writing, and read and approved the final manuscript.

## Supplementary Material

Additional file 1 **Solanaceae fossil record.** Details of all records of Solanaceae with full references to primary sources. Click here for file

Additional file 2 **Solanaceae time-calibrated phylogeny with tips.** A detailed dated phylogeny of Solanaceae showing mean node ages and 95% confidence intervals for all nodes. Posterior probability branch support values are indicated in branch colours, where red refers to nodes with < 80% support. Major clades are indicated, and studies which include more detailed phylogenies of the particular groups are indicated on the left. These studies should be referred to as primary phylogenetic sources for the particular clades with more up-to-date details of species-level relationships because the individual studies used more markers and discuss specific issues relevant at such low taxonomic levels, including polyploidy, hybridisation, and gene tree – species tree incongruences. Click here for file

Additiona file 3 **Solanaceae time-calibrated phylogeny.** Dated molecular phylogeny of the Solanaceae based on the supermatrix calibrated using fossil data. Major clades are shown with their associated ages and 95% confidence intervals. Thick branches indicate highly supported clades with > 0.9 posterior probability. Clade size is proportional to the number of species sampled in each clade. Associated floral and fruit forms are shown on the right. A more detailed view of this phylogeny is shown in Additional file 2. Click here for file

Additional file 4 **New sequence data.** Voucher data and GenBank numbers for sequences newly generated as part of the study. Click here for file

Additional file 5 **New sequence data.** Details of sequences downloaded from GenBank which appeared clearly misidentified or potentially contaminated sequences based on BLAST searches and their position in our preliminary Maximum Likelihood phylogenies. Click here for file

Additional file 6 **Supermatrix sequence alignment file (RAxML input).** Final supermatrix alignment file in phylip format used as input for the Maximum Likelihood phylogeny estimation in RAxML. Click here for file

Additional file 7 **Supermatrix partitions (RAxML input).** Details of the partitioning scheme used in the supermatrix for the Maximum Likelihood analysis in RAxML. Click here for file

Additional file 8 **Supermatrix excluded regions (RAxML input).** Details of the excluded regions within the supermatrix alignment file in RAxML. Click here for file

Additional file 9 **BEAST xml input file.** Input file used for Bayesian relaxed molecular clock dating analysis in BEAST. Click here for file

Additional file 10 **PATHd8 input file.** Input file used for local clock model dating analysis in PATHd8. Click here for file
