# Supplementary material for: A phylogenetic framework for evolutionary study of the nightshades (Solanaceae): a dated 1000-tip tree
Source: BMC Evol Biol. 2013 Sep 30;13:214. doi: 10.1186/1471-2148-13-214 (PMC3850475; doi:10.1186/1471-2148-13-214)
Supplement: Additional file 1 — Solanaceae fossil record. Details of all records of Solanaceae with full references to primary sources. [file 1471-2148-13-214-S1.docx]

# Additional file 1

# Särkinen et al. “A phylogenetic framework for evolutionary study of the nightshades (Solanaceae): a dated 1000-tip tree”

# Table SI1. Full details of all Solanaceae fossils reviewed as part of the study. Fossil ages are derived from bed-level stratigraphic resolution if not otherwise stated. The published geological time scale by Gradstein et al. (2004) was used. Chronostratigraphy was updated for Southern England formations based on King (2006). Specimens in bold were investigated using high-resolution X-ray computed tomography to study fossil identity (T.Särkinen, M.Collinson, P.Kenrick, F.Ahmed, unpublished observations).

| **Name** | **Age (Mya)** | **Epoch** | **Organ** | **Fossil Strata** | **Country** | **Reference** | **Solanaceae** | **Phylogenetic Position here** |
| --- | --- | --- | --- | --- | --- | --- | --- | --- |
| *Datura* cf. *stramonium* | 3.6 - 2.6 | Pliocene: Piazencian | seed | Kholmech, Dnieper river | Belarus | Velichkevich & Zastawniak 2003 | Yes | Solanoideae |
| *Hyoscyamus* sp. | 5.3 - 2.6 | Pliocene | seed | Kroscienko | Poland | Szafer 1946 | Yes | Solanoideae |
| *Hyoscyamus niger* | 3.6 - 2.6 | Pliocene: Piazencian | seed | Pont-de-Gail, Cantal | France | Reid 1923 | Yes | Solanoideae |
| *Physalis alkekengi* | 7.3 - 3.6 | Mio/Pliocene: Messinian-Zanclean | seed | Saugbagger Flora | France | Geissert et al 1990 | Yes | Solanoideae |
| *Physalis alkekengi* | 3.6 - 2.6 | Pliocene: Piazencian | seed | Kholmech, Dnieper river | Belarus | Velichkevich & Zastawniak 2003 | Yes | Solanoideae |
| *Physalis alkekengi* | 3.6 - 2.6 | Pliocene: Piazencian | seed | Bezirk Halle | Germany | Mai & Walther 1988 | Yes | Solanoideae |
| *Physalis pliocenica* | 28.4 - 23.0 | Oligocene: Chattian | seed | Zentendorf | Germany | Mai 1997 | Yes | Solanoideae |
| *Physalis pliocenica* | 5.3 - 2.6 | Pliocene | seed | Kroscienko | Poland | Szafer 1946 | Yes | Solanoideae |
| *Physalis* *pliocenica* | 11.6 - 7.3 | Miocene: Tortonian | seed | Stare Gliwice | Poland | Szafer 1961 | Yes | Solanoideae |
| *Physalis pliocenica* | 13.7 - 11.6 | Miocene: Serravallian | seed | Tongrube Forst Neukollm | Germany | Mai 2001 | Yes | Solanoideae |
| *Physalis pliocenica* | 13.7 - 11.6 | Miocene: Serravallian | seed | Klettwitz | Germany | Mai 2001 | Yes | Solanoideae |
| *Physalis alkekengi* | 7.3 - 3.6 | Mio/Pliocene: Messinian-Zanclean | seed | Nochten-Ost | Germany | Mai 2001 | Yes | Solanoideae |
| *Physalis* aff. *alkekengi* | 5.3 - 3.6 | Pliocene: Zanclean | seed | Baldevo Formation | Bulgaria | Palamarev 1970 | Yes | Solanoideae |
| *Scopolia carniolica* | 3.6 - 2.6 | Pliocene: Piazencian | seed | Bezirk Halle | Germany | Mai & Walther 1988 | Yes | Solanoideae |
| *Solanispermum reniforme* | 48.0 - 46.0 | Eocene: Late Ypresian/Early Lutetian | seed | Lower Bagshot (Arne), Poole Formation | UK | Chandler 1962 | Yes | Solanaceae |
| ***Solanispermum reniforme*** | **48.0 - 46.0** | **Eocene: Late Ypresian/Early Lutetian** | **seed** | **Bournemouth Freshwater Beds, Poole Formation** | **UK** | **Chandler 1963** | **Yes** | **Solanaceae** |
| *Solanispermum reniforme* | 44.0 - 40.0 | Eocene: Late Lutetian | seed | Boscombe Sand Formation | UK | Chandler 1963 | Yes | Solanaceae |
| *Solanispermum reniforme* | 40.4 - 37.2 | Eocene: Bartonian | seed | Highcliffs sands/Cliff End Beds, Barton Formation | UK | Chandler 1963 | Yes | Solanaceae |
| *Solanispermum reniforme* | 33.9 - 28.4 | Oligocene: Rupelian | seed | Bovey Tracey | UK | Chandler 1957 | Yes | Solanaceae |
| *Solanum arnense* | 48.0 - 46.0 | Eocene: Late Ypresian/Early Lutetian | seed | Lower Bagshot (Arne), Poole Formation | UK | Chandler 1962; | Yes | Solanaceae |
| *Solanum* cf. *persicum* | 3.6 - 2.6 | Pliocene: Piazencian | seed | Kholmech | Belarus | Velichkevich & Zastawniak 2003 | Yes | Solanoideae |
| *Solanum dulcamara* | 7.3 - 3.6 | Mio/Pliocene: Messinian-Zanclean | seed | Saugbagger Flora | France | Geissert et al 1990 | Yes | Solanoideae |
| *Solanum dulcamara* | 3.6 - 2.6 | Pliocene: Piazencian | seed | Bezirk Halle | Germany | Mai & Walther 1988 | Yes | Solanoideae |
| *Solanum dulcamara* | 3.6 - 2.6 | Pliocene: Piazencian | seed | Nordhausen | Germany | Mai & Walther 1988 | Yes | Solanoideae |
| *Solanum dulcamara* | 3.6 - 2.6 | Pliocene: Piazencian | seed | Rippersroda | Germany | Mai & Walther 1988 | Yes | Solanoideae |
| *Solanum dulcamara* | 5.3 - 2.6 | Pliocene | seed | Tegelen-Sur-Meuse | Holland | Reid & Reid 1907 | Yes | Solanoideae |
| *Solanum dulcamara* | 3.6 - 2.6 | Pliocene: Piazencian | seed | Pont-de-Gail, Cantal | France | Reid 1920 | Yes | Solanoideae |
| *Solanum dulcamara* | 3.6 - 2.6 | Pliocene: Piazencian | seed | Pont-de-Gail, Cantal | France | Reid 1923 | Yes | Solanoideae |
| *Solanum dulcamara* | 5.3 - 2.6 | Pliocene | seed | Kroscienko | Poland | Szafer 1946 | Yes | Solanoideae |
| *Solanum nigrum* | 11.6 - 5.3 | Miocene: Tortonian-Messinian | seed | North Rhine, Hambach | Germany | Van der Burgh 1987 | Yes | Solanoideae |
| *Solanum* sp. | 5.3-2.6 | Pliocene | seed | Limburg & Prussian border | Holland | Reid & Reid 1915 | Yes | Solanoideae |
| ***Cantisolanum daturoides*** | **55.0 – 50.0** | **Eocene: early Ypresian** | **seed** | **London Clay Formation** | **UK** | **Reid & Chandler 1933** | **No** | **-** |
| Unknown Solanaceae | 5.3-2.6 | Pliocene | seed | Limburg & Prussian border | Holland | Reid & Reid 1915 | No | - |
| *Physalis* sp. | 5.3 - 0.0 | Plio/Pleistocene | seed | Torre Picchio section (P11 & P12), Santa Maria di Ciciliano Formation | Italy | Girotti et al. 2003 | ? ^1^ | - |
| *Solanum* sp. | 1.7 - 1.2* | Pleistocene | seed | Olduvai Gorge | Tanzania | Tobias 1991 | ? ^1^ | - |
| *Physalis* sp. | 23.0 - 16.0 | Miocene: Aquitanian-Burdigalian | seed | Kireevsky Village, Ob River | Russia | Dorofeev 1963 | ? ^2^ | - |
| *Solanum* sp. #1 | 3.6 - 2.6 | Pliocene: Piacenzian | seed | Chernoluch Village, Irtysh River | Russia | Dorofeev 1963 | ? ^2^ | - |
| *Solanum* sp. #2 | 16.0 - 11.6 | Miocene: Langhian-Serravallian | seed | Novonikolsky Village, Irtysh River | Russia | Dorofeev 1963 | ? ^2^ | - |
| *Solanum* sp. #2 | 16.0 - 11.6 | Miocene: Langhian-Serravallian | seed | Ebargulsky Village, Irtysh River | Russia | Dorofeev 1963 | ? ^2^ | - |
| *Solanumxylon* *paranensis* | 16.0 - 11.6 | Miocene: Langhian-Serravallian | wood | Paraná Formation | Argentina | Franco & Brea 2008 | Yes | Solanaceae |
| Solanaceae or Asteraceae | 70.6 - 65.5 | Cretaceous: Maastrichtian | wood | Panoche Formation, Del Puerto | USA | Page 1980 | No | - |
| Pollen forma C | 72.0 - 68.0 | Cretaceous: Campanian-Maastrichtian boundary | pollen | San Joaquin Valley (D-1 & D-2), Great Valley Sequence | USA | Chmura 1973 | No | - |
| *Datura* cf. *discolor* | 37.2 - 33.9 | Eocene: Priabonian | pollen | Florissant Basin | USA | Leopold & Clay-Poole 2001 | ? ^1^ | - |
| *Solanum* sp. | 5.3 - 0.0 | Plio/Pleistocene | pollen | Olduvai Gorge | Tanzania | Hay 1976 | ? ^1^ | - |
| *Solandra haeliadum* | 55.8 -33.9 | Eocene | leaf | Salcedo | Italy | Massalongo 1851 | No | - |
| *Solanites brongniartii* | 33.9 - 23.0 | Oligocene | flower | Aix-en-Provence | France | Saporta 1862 | No | - |
| *Solanites crassus* | 55.8 - 40.4 | Eocene: Lutetian-Ypresian | flower | Claiborne | USA | Berry 1930 | No | - |
| *Solanites pusillus* | 55.8 - 40.4 | Eocene: Lutetian-Ypresian | flower | Claiborne | USA | Berry 1930 | No | - |
| *Solanites saportanus* | 55.8 - 40.4 | Eocene: Lutetian-Ypresian | flower | Claiborne | USA | Berry 1916 | No | - |
| *Solanites sarachaformis* | 55.8 - 40.4 | Eocene: Lutetian-Ypresian | flower | Claiborne | USA | Berry 1930 | No | - |

^1^ No description nor illustration or proper reference to specimen

^2^ Reference not located

* Radiometric dating

**Authors’ notes and observations on fossil-taxa**

*Cantisolanum daturoides*: Seeds obovate, flattened; hilum at top; embryo obovate

*Datura* cf. *stramonium:* Seeds reniform, 3.9 mm long, 2.8 mm wide, flattened; hilar-chalazal cavity present; hilum sub-lateral; testa cells slightly digitate; embryo curved

*Hyoscyamus* sp.: Seeds sub-reniform, 2.4-2.8 mm long, 1.7-2.1 mm wide, strongly flattened; hilum lateral; testa cells digitate, large, with raised cell walls; embryo shape unclear

*Hyoscyamus niger:* Seeds circular, strongly flattened; hilum marginal; testa cells honey-combed shaped, large, with raised cell walls; embryo shape unclear

*Physalis alkekengi*: Seeds obovate to circular, 1.7-2.2 mm long, 1.8-2.5 mm wide, flattened; hilum sub-lateral; testa cells digitate; embryo curved

*Physalis pliocenica*: Seeds circular to reniform, 1.7-1.9 mm long, 1.3-1.6 mm wide, flattened; hilum sub-central to lateral; testa cells digitate; embryo curved

*Scopolia carniolica:* Seeds ovate, 2.4-2.8 mm long, 1.8-2.1 mm wide, flattened; testa cells digitate, large, with raised cell walls; embryo curved

*Solandra haeliadum:* Non-specific leaf characters presented

*Solanispermum reniforme*: Seeds reniform, flattened; hilum gaping, sub-centrally positioned; hilar-chalazal cavity present; embryo curved

*Solanites brongniartii:* Corolla pentamerous, rotate; anthers long, longitudinally dechiscent with connective projections, filaments short; styles two, stigmas two, capitate

*Solanites crassus:* Non-specific characters: corolla stellate, with five petals united at base; stamens five, free; anthers longitudinally dehiscent

*Solanites pusillus:* Non-specific characters: corolla stellate, with five petals united at base; stamens five, free; anthers longitudinally dehiscent

*Solanites saportanus:* Non-specific characters: corolla stellate, with five petals united at base; stamens five, free; anthers longitudinally dehiscent

*Solanites sarachaformis:* Non-specific characters: corolla stellate, with five petals united at base; stamens five, free; anthers longitudinally dehiscent

*Solanum* aff. *nigrum:* Seed coat, 1.5 mm long, 1.5 mm wide, sub-circular in shape; hilum central; testa cells digitate; embryo curved

*Solanum arnense*: Hilum sub-centrally positioned, gaping; hilar-chalazal cavity present; testa cells digitate; embryo curved

*Solanum* cf. *persicum:* Seeds reniform, 1.5-1.9 mm long, 2.2-2.4 mm wide, flattened; hilum lateral; testa cells digitate; embryo curved

*Solanum dulcamara*: Seeds reniform, 1.7-1.8 mm long, 1.2-2.7 mm broad, circulate to ovate, flattened; hilum central to lateral; testa cells digitate, cell walls somewhat raised; embryo curved

*Solanum nigrum:* Seeds circular, 1.5 mm wide, flattened; hilum lateral; testa cells digitate; embryo curved

*Solanum* sp.: Seeds circular, 1.8 mm long, 1.8 mm wide, flattened; hilum sub-lateral; testa cells digitate; embryo curved

*Solanumxylon* *paranensis*: Various (listed in Franco & Brea 2008); detailed comparison made to extant *Solanum* species from the area where fossil discovered, not across the genus. Placement of the fossil in the stem node of *Solanum* could be justified.

Unknown Solanaceae (Reid & Reid 1915): Seeds circular, 1.8 mm long, flattened; testa cells quadrate or hexagonal; embryo shape unclear

Pollen forma C: Pollen grains 3-colporate, 5-colpate, prolate shaped, with striate ornamentation; Same characters found in *Lycium*, *Nolana*, and *Hyoscyamus* but also outside Solanaceae in *Brucea* (Simaroubaceae) and *Skimmia* (Rutaceae), and hence the pollen is not assigned to Solanaceae here

**References**

Berry EW 1916 The lower Eocene floras of Southeastern North America. U.S. Geological Survey Professional Paper 91: 1-481

Berry EW 1930 Revision of the lower Eocene Wilcox flora of the southeastern States, with descriptions of new species, chiefly from Tennessee and Kentucky. U.S. Geological Survey Professional Paper 156: 1-196

Chandler MEJ 1962 The Lower Tertiary Floras of Southern England II Flora of the Pipe-Clay Series of Dorset (Lower Bagshot). British Museum, London.

Chandler MEJ 1963 The Lower Tertiary Floras of Southern England. III. Flora of the Bournemouth Beds, The Boscombe, and the Highcliff Sands. British Museum, London.

Chandler MEJ 1957 The Oligocene Flora of the Bovey Tracey Lake Basin, Devonshire. Bulletin of the British Museum (Natural History) Geology 3:71-123

Chmura CA 1973 Upper Cretaceous (Campanian-Maastrichtian) angiosperm pollen from the western San Joaquin Valley, California, U.S.A. Palaeontographica Abt B 141: 89-171.

Collinson ME, Boulter MC, Holmes PL 1993 Magnoliophyta (“Angiopsermae”). In The Fossil Record 2, ed by MJ Benton 809-841. Chapman & Hall, London.

Dorofeev PI 1963. *Tretichnye Flory Zapadnoi Sibiri (Tertiary Floras of Western Siberia). Izdat. Akad. Nauk, Moscow/Leningrad.*

Franco MJ and Brea M 2008 Leños fósiles de la formación Paraná (Mioceno Medio), Toma Vieja, Paraná, Entre Ríos, Argentina: registro de bosques estacionales mixtos. Ameghiniana 45: 699-717

Geissert F, Gregor HJ, Mai DH, Boenigk W, Guent T 1990 Die “Saugbaggerflora”, eine Frucht- und Samenflora aus dem Grenzbereich Miozaen- Pliozaen von Sessenheim im Elsass (Frankreich). Documenta naturae 57: 1-207

Girotti O, Barbato C, Esu D, Gliozzi E, Kotsakis T, Martinetto E, Petronio C, Sardella R, and Squazzini E 2003. The section of Torre Picchio (Terni, Umbria, Central Italy): A Villafranchian site rich in vertebrates, molluscs, ostracods and plants. Rivista Italiana di Paleontologia e Stratigrafia 109: 77-98

Gradstein F, Ogg J, Smith AG 2004 A geologic time scale 2004. Cambridge University Press, Cambridge, UK

Hay RL 1976 Geology of the Olduvai Gorge. Univeresity of California Press, Berkeley

King C 2006 Paleogene and Neogene: Uplift and a cooling climate. In “The geology of England and Wales”, 2^nd^ edition, PJ Brenchley & PF Rawson (eds.), pp. 395-428, The Geological Society, London

Leopold EB, Clay-Poole ST 2001 Florissant leaf and pollen floras of Colorado compared; climatic implications. Pages 17-69 in E Evanoff, G Wodzicki, M Kathrun, and KR Johnson. Fossil flora and stratigraphy of the Florissant Formation, Colorado. Proceedings of the Denver Museum of Natural History 4, no. 1.

Mai DH & Walther H 1988 Die Pliozaenen Floren von Thueringen, Deutsche Demokratische Republik. Quartaerpalaeontologie 7: 55-297

Mai DH 1997 Die oberoligozanen Floren am Nordrand der Sachsischen Lausitz. Palaeontographica Abteilung B 244: 1-124

Mai DH 2001 Die mittelmiozaenen und obermiozaenen Floren aus der Meuroer und Raunoer Folge in der Lausitz. III. Fundstellen und Palaeobiologie. Palaeontographica Abteilung B 258:1-85

Martínez-Millan M 2010 Fossil record and age of the Asteridae. Botanical Review 76: 83-135

Massalongo A 1851 Sopra le piante fossili dei terreni terziarj del Vicentino. A. Bianchi Padova.

Page VM 1980 Dicotyledonous wood from the Upper Cretaceous of central California II. J. Arnold Arb 61: 723-748

Palamarev E 1970 Fossile Floren aus drei Braun kohlebecken in Sudwestbulgarien. Izv. Bot. Inst. (Sofia) 20: 35-79

Palamarev E, Bozukov V, Uzunova K, Petkova A, Kitanov G 2005 Catalogue of the Cenozoic plants of Bulgaria (Eocene to Pliocene). Phytologia Balcanica 11: 215-364

Reid C & Chandler MEJ 1933 The London Clay Flora. British Museum (Natural History), London.

Reid C & Reid EM 1907 The fossil flora of Tegelen-sur-Meuse, near Venloo, in the province of Limburg. Verhandelingen der Koninklijke Akademie van Wetenschappen, Afdeeling natuurkunde, Tweedie sectie 13: 1-27

Reid C & Reid EM 1915 The Pliocene Floras of the Dutch-Prussian border. Mededeelingen van de Rijksopsporing van Delfstoffen, The Hague.

Reid EE 1920 Recherches sur quelques graines Pliocenes du Pont-de-Gail (Cantal). Bull Soc. Geol. De France Ser. IV 20: 48-79

Reid EM 1923 Nouvelles recherches sur les graines Pliocene inferieur du Pont-de-Gail (Cantal). Bull. Soc. Geol. France Ser. IV 23: 308-355

Särkinen T, Collinson M, Kenrick P, Ahmed F 2013 Using high-resolution X-ray computed tomography to study small fossil seeds in Solanaceae. *PhytoKeys*, in preparation.

Szafer W 1946 Flora Pliocenska z Kroscienska n dunajcem (The Pliocene Flora of Kroscienko in Poland). Polska Akademia Umiej etnosci, Krakow.

Szafer W 1961 Miocenska Flora ze Starych Gliwic na Slasku (Miocene Flora of Stare Gliwice in Upper Silesia). Prace Instytut Geologiczny 33: 1-205

Tobias PV 1991 Olduvai Gorge, parts I-IV: The skulls, endocasts, and teeth of Homo habilis. Cambridge University Press, Cambridge

Van der Burgh J 1987 Miocene floras in the Lower Rhenish basin and their ecological interpretation. Review of Palaeobotany and Playnology 52: 299-366

Velichkevich FY & Zastawniak E 2003 The Pliocene flora of Kholmech, south-eastern Belarus and its correlation with other Pliocene floras Europe. Acta Paleobot. 43: 137-259
