# Supplementary figures and images for: A phylogenetic framework for evolutionary study of the nightshades (Solanaceae): a dated 1000-tip tree
Source: BMC Evol Biol. 2013 Sep 30;13:214. doi: 10.1186/1471-2148-13-214 (PMC3850475; doi:10.1186/1471-2148-13-214)

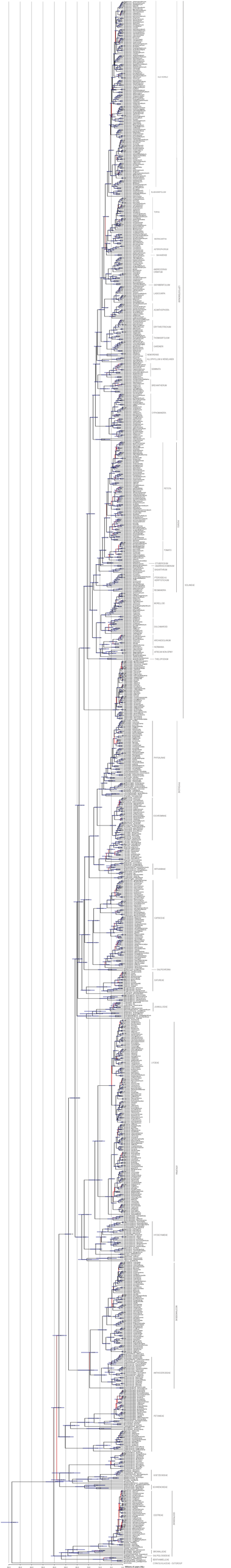

Supplement: Additional file 2 — Solanaceae time-calibrated phylogeny with tips. A detailed dated phylogeny of Solanaceae showing mean node ages and 95% confidence intervals for all nodes. Posterior probability branch support values are indicated in branch colours, where red refers to nodes with < 80% support. Major clades are indicated, and studies which include more detailed phylogenies of the particular groups are indicated on the left. These studies should be referred to as primary phylogenetic sources for the particular clades with more up-to-date details of species-level relationships because the individual studies used more markers and discuss specific issues relevant at such low taxonomic levels, including polyploidy, hybridisation, and gene tree – species tree incongruences. [file 1471-2148-13-214-S2.tiff]

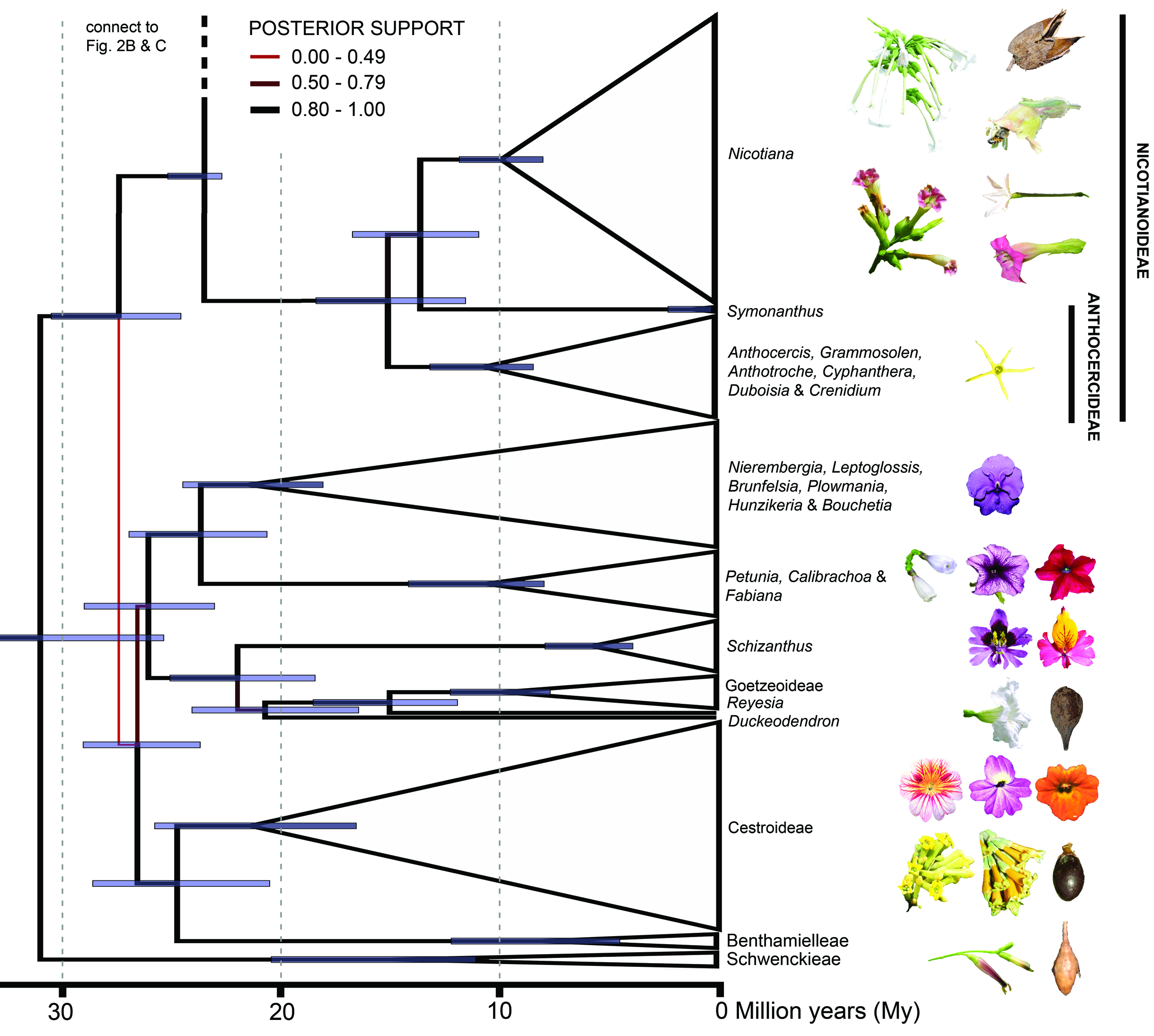

Supplement: Additiona file 3 — Solanaceae time-calibrated phylogeny. Dated molecular phylogeny of the Solanaceae based on the supermatrix calibrated using fossil data. Major clades are shown with their associated ages and 95% confidence intervals. Thick branches indicate highly supported clades with > 0.9 posterior probability. Clade size is proportional to the number of species sampled in each clade. Associated floral and fruit forms are shown on the right. A more detailed view of this phylogeny is shown in Additional file 2. [file 1471-2148-13-214-S3.zip › figure 3/8730481359552793_fig3_bottom.tiff]

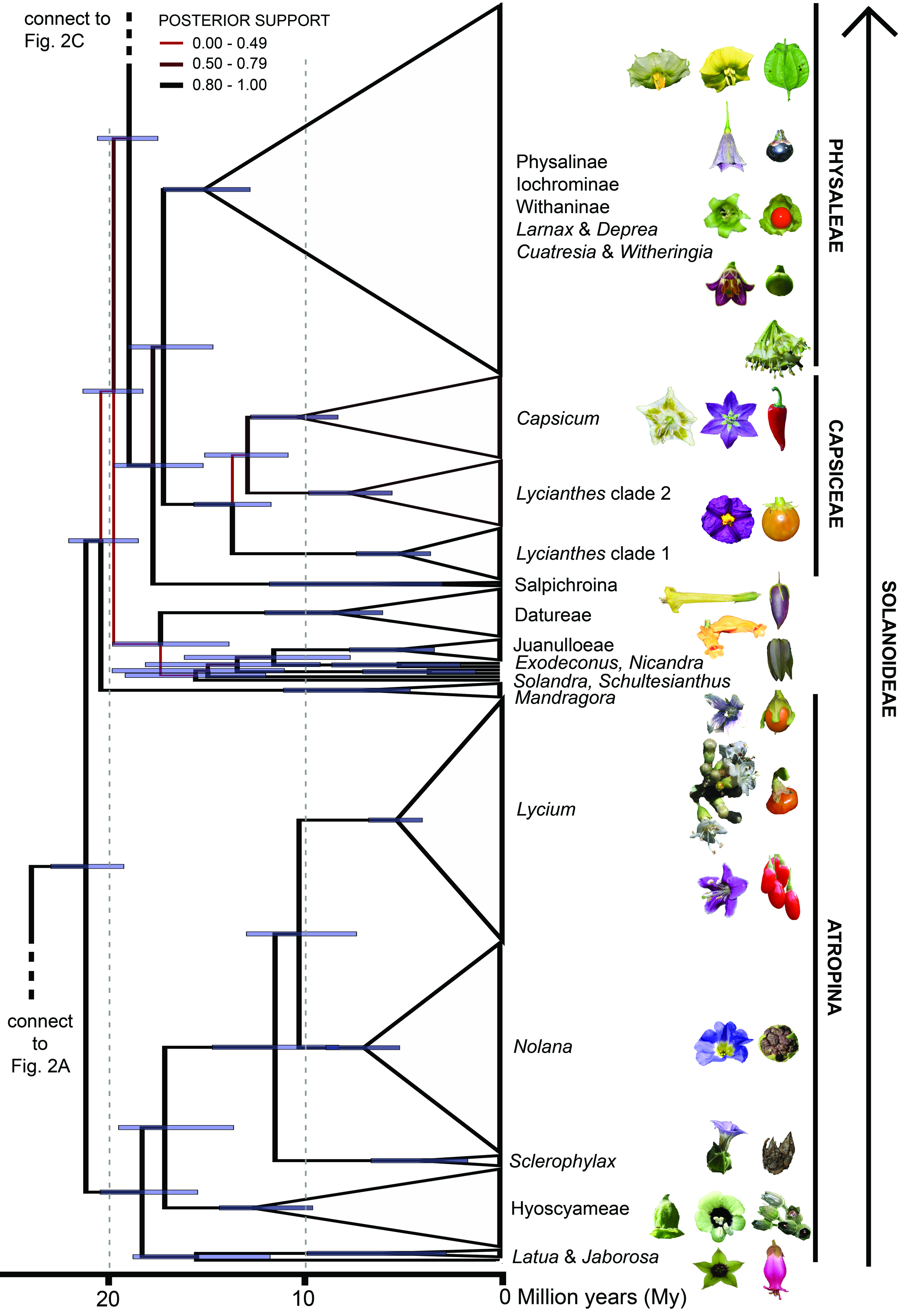

Supplement: Additiona file 3 — Solanaceae time-calibrated phylogeny. Dated molecular phylogeny of the Solanaceae based on the supermatrix calibrated using fossil data. Major clades are shown with their associated ages and 95% confidence intervals. Thick branches indicate highly supported clades with > 0.9 posterior probability. Clade size is proportional to the number of species sampled in each clade. Associated floral and fruit forms are shown on the right. A more detailed view of this phylogeny is shown in Additional file 2. [file 1471-2148-13-214-S3.zip › figure 3/8730481359552793_fig3_middle.tiff]

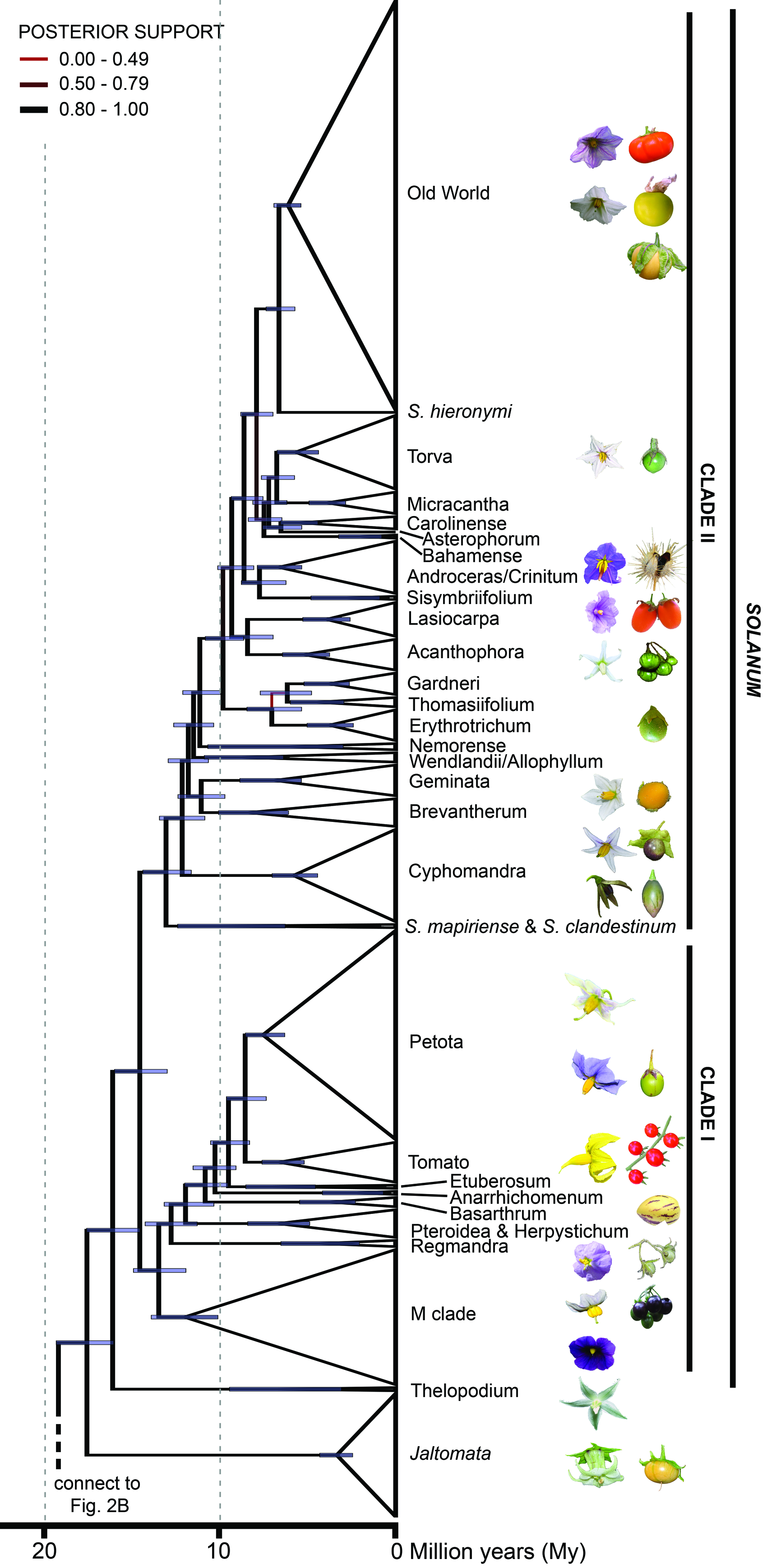

Supplement: Additiona file 3 — Solanaceae time-calibrated phylogeny. Dated molecular phylogeny of the Solanaceae based on the supermatrix calibrated using fossil data. Major clades are shown with their associated ages and 95% confidence intervals. Thick branches indicate highly supported clades with > 0.9 posterior probability. Clade size is proportional to the number of species sampled in each clade. Associated floral and fruit forms are shown on the right. A more detailed view of this phylogeny is shown in Additional file 2. [file 1471-2148-13-214-S3.zip › figure 3/8730481359552793_fig3_top.tiff]
