# Supplementary material for: A phylogenetic framework for evolutionary study of the nightshades (Solanaceae): a dated 1000-tip tree
Source: BMC Evol Biol. 2013 Sep 30;13:214. doi: 10.1186/1471-2148-13-214 (PMC3850475; doi:10.1186/1471-2148-13-214)
Supplement: Additional file 4 — New sequence data. Voucher data and GenBank numbers for sequences newly generated as part of the study. [file 1471-2148-13-214-S4.docx]

| **Species** | **Voucher** | **DNA #** | **GenBank number** | | | | |
| --- | --- | --- | --- | --- | --- | --- | --- |
|  |  |  | ***trnS-G*** | ***waxy*** | ***ndhF*** | ***trnL-F*** | **ITS** |
| *Athenaea pogogena* (Moric.) Sendtn. | Agra et al. 7177 (JPB) | LB2690 |  |  | x | x | x |
| *Benthamiella patagonica* Speg. | Moree 2563 (K) | - |  |  | x | x |  |
| *Deprea sylvarum* (Standl. & C.V. Morton) Hunz. | Lepiz 566 (BM) | - |  |  | x | x |  |
| *Heteranthia decipiens* Nees & Mart. | Agra 7184 (JPB) | LB2676 |  |  | x | x |  |
| *Archihyoscyamus leptocalyx* (Stapf) A.M. Lu | Davis 28330 (BM) | - |  |  | x | x |  |
| *Metternichia* sp. | Agra 7199 (JPB) | LB2686 |  |  |  |  | x |
| *Reyesia* sp. | Wood 21349 (K) | - |  |  |  | x |  |
| *Solanum guineense* L. | Henekom 2964 (NBG) | RGO99-25 |  |  |  |  | x |
| *Solanum batoides* D'Arcy & Rakot. | Ronaijojaone et al. 130 (P) | LB2533 |  |  |  | x | x |
| *Solanum capsiciforme* (Domin) G.T.S.Baylis | 884750213 (NIJ) | LB710 |  |  |  |  | x |
| *Solanum clandestinum* Bohs | Nee 51781 (NY) | LB779 |  |  |  |  | x |
| *Solanum enantiophyllanthum* Bitter | Flinte s.n. (UT) | LB2152 | x | x |  |  |  |
| *Solanum monarchostemon* S.Knapp | Gentry et al. 37156 (MO) | - |  | x |  | x |  |
| *Solanum multifidum* Lam. | Bennett 103 (BM) | LB1968 |  | x |  |  | x |
| *Solanum nakurense* C.H.Wright | Vorontsova et al. 56 (BM) | LB2483 |  |  |  | x | x |
| *Solanum pallidum* Rusby | Nee 51759 (NY) | LB787 |  |  | x |  |  |
| *Solanum paposanum* Phil. | Aedo 10976 (MA) | LB2000 |  | x |  | x | x |
| *Solanum reductum* C.V.Morton | Barboza et al. 1955 (CORD) | LB2353 | x | x |  |  |  |
| *Solanum riojense* Bitter | Nee & Bohs 50843 (NY) | LB544 | x |  |  |  |  |
| *Solanum simile* F.Muell. | Bohs 2458 (UT) | LB102 |  | x |  |  | x |
| *Solanum symonii* H.Eichler | Symon 446 (AD) | LB2333 |  | x |  |  | x |
| *Solanum terminale* Forssk. | Vorontsova et al. 93 (BM) | LB2493 |  |  |  | x | x |
| *Solanum tredecimgranum* Bitter | Barboza 2399 (CORD) | - |  |  | x |  |  |
| *Solanum triflorum* Nutt. | Bohs 3062 (UT) | LB861 | x |  |  |  |  |
| *Solanum valdiviense* Dunal | Gardner & Knees 6726 (BM) | LB2756 |  | x |  | x | x |
| *Solanum vescum* F.Muell. | BIRM S.0800 (NIJ) | RGO298 |  |  |  |  | x |
| *Trianaea* sp. | Lewis et al. 2945 (BM) | - |  |  | x | x |  |
| *Trianaea speciosa* (Drake) Soler. | Lewis et al. 3250 (BM) | - |  |  |  | x |  |

# Additional file 3

# Särkinen et al. “A phylogenetic framework for evolutionary study of the nightshades (Solanaceae): a dated 1000-tip tree”

**Table SI2.** Voucher data for species for which new sequence data was generated. LB refers to Lynn Bohs and RGO to Richard Olmstead extractions.
