# Supplementary material for: A phylogenetic framework for evolutionary study of the nightshades (Solanaceae): a dated 1000-tip tree
Source: BMC Evol Biol. 2013 Sep 30;13:214. doi: 10.1186/1471-2148-13-214 (PMC3850475; doi:10.1186/1471-2148-13-214)
Supplement: Additional file 5 — New sequence data. Details of sequences downloaded from GenBank which appeared clearly misidentified or potentially contaminated sequences based on BLAST searches and their position in our preliminary Maximum Likelihood phylogenies. [file 1471-2148-13-214-S5.docx]

| **GenBank number** | **Region** | **GenBank ID** | **Closest sequence identity & placement in phylogeny** |
| --- | --- | --- | --- |
| EF438980 | matK | *Physalis* sp. | *Nicandra* |
| EF438857 | matK | *Physalis* sp. | *Nicandra* |
| AY028148 | ITS2 | *Jaborosa integrifolia* | *Nolana* |
| AY028130 | ITS1 | *Jaborosa integrifolia* | *Nolana* |
| AF244710 | ITS | *Jaltomata procumbens* | *Iochroma* |
| AB019288 | ITS1 | *Atropa belladonna* | *Anisodus* |
| AY028129 | ITS1 | *Atropa belladonna* | *Anisodus* |
| AY028147 | ITS2 | *Atropa belladonna* | *Anisodus* |
| AB019948 | ITS2 | *Atropa belladonna* | *Anisodus* |
| AF244709 | ITS | *Lycianthes heteroclita* | *Solanum* |
| DQ314160 | ITS | *Salpichroa tristis* | *Nicotiana* |
| AY478412 | ITS1 | *Scopolia japonica* | *Solanum* |
| AY478398 | ITS1 | *Scopolia japonica* | *Solanum* |
| AY478412 | ITS2 | *Scopolia japonica* | *Solanum* |
| AY478412 | ITS2 | *Scopolia japonica* | *Solanum* |
| GU591063 | ITS | *Solanum buddleifolium* | *Solanum viarum*, but with only 85% sequence similarity |
| AY996543 | ITS | *Solanum polygamum* | *Chamaesaracha* |

# Additional file 4

# Särkinen et al. “A phylogenetic framework for evolutionary study of the nightshades (Solanaceae): a dated 1000-tip tree”

**Table S4.** Details of sequences downloaded from GenBank but which were excluded from our analyses due to suspected erroneous voucher identification or sample contamination. Closest sequence identity was derived from BLAST searches, but sequence placement in the Maximum Likelihood topology was used to detect the questionable accessions.
